# Decreased IL-4 is the risk factor of depression in patients with Takayasu arteritis

**DOI:** 10.3389/fmed.2024.1337206

**Published:** 2024-02-15

**Authors:** Yaxin Zhang, Shiyu Yang, Anyuyang Fan, Juan Du, Na Gao, Lili Pan, Taotao Li

**Affiliations:** ^1^Department of Rheumatology and Immunology, Beijing Anzhen Hospital, Capital Medical University, Beijing, China; ^2^Department of the National Clinical Research Center for Mental Disorders and Beijing Key Laboratory of Mental Disorders, Beijing Anding Hospital and the Advanced Innovation Center for Human Brain Protection, Capital Medical University, Beijing, China

**Keywords:** Takayasu arteritis, mental psychology, depression, cytokines, disease activity

## Abstract

**Objective:**

Depression is a common complication in Takayasu arteritis (TA). Disorders of the immune system play an important role in both diseases. This study aimed to clarify the feature of cytokines in TA patients with depression.

**Methods:**

In this cross-sectional study, serum cytokines were tested in 40 TA patients and 11 healthy controls using the Bio-Plex Magpix System (Bio-Rad^®^). The state of depression was measured by the Zung Self-Rating Depression Scale (SDS) in TA patients. Logistic regression analysis was performed to find the risk factors of depression in patients with TA.

**Results:**

TA patients with depression had higher ESR, hsCRP, NIH, and ITAS.A than patients without depression (16.00 [10.00, 58.50]mm/H vs. 7.50 [4.50, 17.75]mm/H, *p* = 0.013; 7.60 [2.32, 46.52]mg/L vs. 0.71 [0.32, 4.37]mg/L, *p* = 0.001; 2.00 [2.00, 3.00] vs. 1.00 [0.00, 2.00], *p* = 0.007; 7.00 [4.00, 9.50] vs. 1.50 [0.00, 5.75], *p* = 0.012, respectively). Additionally, the lower age of onset and levels of IL-4, IL-13, eotaxin, and IP-10 were observed in the depressed group compared with the non-depressed (23.50 [19.25, 32.50]pg./ml vs. 37.00 [23.25, 42.50]pg./ml, *p* = 0.017; 2.80 [2.17, 3.18]pg./ml vs. 3.51 [3.22, 4.66]pg./ml, *p* < 0.001; 0.66 [0.60, 1.12]pg./ml vs. 1.04 [0.82, 1.25]pg./ml, *p* = 0.008; 46.48 [37.06, 61.75]pg./ml vs. 69.14 [59.30, 92.80]pg./ml, *p* = 0.001; 184.50 [138.23, 257.25]pg./ml vs. 322.32 [241.98, 412.60]pg./ml, *p* = 0.005, respectively). The lower level of IL-4 and age of onset were the independent risk factors for depression in TA patients (OR [95% CI] 0.124 [0.018, 0.827], *p* = 0.031; 0.870 [0.765, 0.990], *p* = 0.035, respectively).

**Conclusion:**

Our data suggested that lower cytokine levels, especially IL-4, might be involved in the development of TA patients with depression. Clinicians can probably use serum IL-4 level testing as a potential indicator of depression in TA.

## Introduction

Takayasu arteritis (TA) is a chronic inflammatory vasculitis that affects the aorta and its major branches, predominantly occurring in young women in 80–90% of cases (1). Inflammation within the vasculature can result in segmental stenosis, occlusion, dilatation, and/or aneurysm formation (2). Existing evidence strongly indicates that cell-mediated immunity is a crucial factor in the pathogenic cascade leading to these lesions (3, 4). However, the quantitative and qualitative changes in cytokine production observed in TA remain incompletely elucidated in current studies.

Pathophysiological investigations into depression have elucidated a strong association between neuroimmune inflammatory pathways and the cytokine-mediated activation of cellular immunity (5). Individuals with major depression often manifest elevated levels of inflammatory mediators, including cytokines, chemokines, and acute-phase proteins in blood or cerebrospinal fluid (6). It is proposed that the onset and persistence of depression are substantially influenced by the secretion of peripheral pro-inflammatory cytokines and other factors. This activation initiates localized, low-grade, yet chronic inflammation within the central nervous system (6, 7). It is now recognized that the cytokines themselves are capable of triggering or inhibiting the release of other cytokines (8). Against the background of inflammation, cytokines act beyond the normal regulatory range. The abnormal phenomenon, in turn, leads to an increase in oxidative stress, thus maintaining, even enhancing chronic inflammation till depression happens (9).

TA is a disease caused by a dysregulation of the systemic immune system (10). In previous studies, a certain proportion of TA patients would have developed depressive co-morbidities (11). Though the mechanism of the disease is not fully defined, it is generally believed that the pathogenic process includes disruption of the cytokine network, which may underlie the pathophysiology of combined depression in TA (12). Previous studies have identified dysregulation of pro- and anti-inflammatory cytokines during inflammatory disease progression as an underlying factor in depression (13). However, the cytokine levels in depression TA patients were unclear. In our study, we detected the serum cytokines (27 types) in depression TA patients. The authors aimed to clarify the characteristics and risk factors of depression in TA patients and provided new evidence for the clinical manifestations and mechanisms of such co-morbidities in the context of systemic inflammatory states.

## Materials and methods

### Patients

A cross-sectional study was carried out to determine whether there is a relationship between cytokines and depression in TA patients. The authors consecutively enrolled 40 hospitalized patients with TA who attended Beijing Anzhen Hospital from March 2021 to September 2022, along with 11 healthy controls recruited from the general population. Cytokine profiles were compared with non-depressed healthy controls to examine the differences between these populations. Clinical and biological data were collected concurrently with the recruitment process of the patient registry.

### Inclusion and exclusion criteria

TA patients who had been treated for more than 6 months were recruited, fulfilling the classification criteria of TA developed by the American College of Rheumatology (ACR)/EULAR in 2022 (14). The following were the exclusion criteria: (1) chronic or current infections, (2) tumors, (3) other autoimmune diseases, and (4) other psychiatric diseases or a history of psychiatric diseases. The authors also included healthy controls with matched age–sex ratios and no previous history of illness, which were evaluated by the SDS to confirm the absence of depressive symptoms.

### Ethics statement

This study was conducted in accordance with the ethical principles Helsinki Declaration and was approved by the Ethics Committee of Beijing Anzhen Hospital (Approval Number: 2023183X).

### Collection of clinical data and laboratory parameters

The authors collected the age of onset, gender, education level, and body mass index (BMI) as demographic variables. Clinical parameters consisted of NIH (15), ITAS.A, and ITAS2010 (16), which were defined to evaluate disease activity and Numano type (17). The authors also recorded in detail the typical clinical symptoms presented during the course of the disease, such as fever, neck pain, chest tightness/chest pain, bilateral pulse inequality, intermittent claudication, dizziness/headache, and abdominal pain (18). All patients were treated with medications, including glucocorticoids, conventional synthetic disease-modifying antirheumatic drugs (cDMARDs), and biologic disease-modifying antirheumatic drugs (btDMARDs). The authors documented the glucocorticoid dosage at the time of enrollment and converted it to prednisone equivalents. The dosages and methods of administration of DMARDs were methotrexate 15 mg Qw, martimecrolide 0.75 g bid, cyclophosphamide 100 mg Qod, hydroxychloroquine sulfate 0.2 g Qd, azathioprine 100 mg Qd, tocilizumab 8 mg/kg Qm, adalimumab 40 mg Q2w, and baricitinib 2 mg Qd. Laboratory parameters included white blood cell count (WBC), neutrophils (NEUT), hemoglobin (Hb), platelets (PLT), erythrocyte sedimentation rate (ESR), and high-sensitivity c-reactive protein (hsCRP), which were assessed at the same time as the estimation of psychological status by the laboratory medicine of Anzhen Hospital.

### Evaluation of depression

Depressive symptoms were evaluated by using the Zung Self-Rating Depression Scale (SDS), a well-established and valid instrument for identifying depression in primary care patients (19). Participants with SDS scores equal to or exceeding 52 were categorized as experiencing depression, while those with SDS scores below 52 were classified as not having depression (20). The healthy controls were also screened for depressive tendencies through the SDS. None reported the presence of depressive symptoms.

### Detection of cytokines

Venous blood (5 mL) was drawn into a vacuum blood collection tube on the day of assessing depressive symptoms. The clotted blood stood for 30 min before centrifugation at 2,000 × g for 10 min at room temperature. The serum was aliquoted and stored in an −80°C freezer for subsequent analysis.

Cytokine levels were determined by the Bio-Plex Pro Human Inflammation and Treg Cytokine Assays (United States) on the Bio-Plex Magpix System (Bio-Rad^®^). The panel analyzed 27cytokines, including IL-1β, IL-1rα, IL-2, IL-4, IL-5, IL-6, IL-7, IL-8, IL-9, IL-10, IL-12(p70), IL-13, IL-15, IL-17, eotaxin, FGF basic, G-CSF, GM-CSF, IFN-γ, IP-10, MCP-1, MIP-1α, MIP-1β, PDGF-BB, RANTES, TNF-α, and VEGF. The samples were processed according to the Bio-Plex Pro Human Inflammation Panel I Assay Quick Guide (Bio-Rad^®^, 10,044,282). The concentrations of the analytes in the test samples were determined by the standard curves of the different concentrations of cytokines.

### Statistical analysis

The demographic and clinical data were expressed as median and IQR as the data results did not conform to normal distribution. The Wilcoxon test was used to determine the significance. The ANOVA test was used to analyze the relationship between the levels of each cytokine profile and the different groups. Spearman’s test was used to determine the correlation between SDS scores, ESR, hsCRP, and cytokines. Multivariate logistic regression analysis was performed to find out the risk factors of TA patients with depression. The analysis and visualization of all statistical tests were exhibited by R version 3.1.1 and SPSS.26.

## Results

### Sociodemographic characteristics and clinical differences in the depressed TA group versus the non-depressed group

In our research, 40 TA patients who had received pharmacological treatment for more than 6 months were recruited. The sociodemographic and clinical data are shown in Table 1; 12 patients with SDS scores ≥52 were categorized as the depressed group and 28 patients with SDS scores lower than 52 were recognized as the non-depressed. The authors found that the age of onset in the depressed group was significantly lower than the non-depressed group in TA patients (23.50 [19.25, 32.50] vs. 37.00 [23.25, 42.50], *p* = 0.017). There were no significant differences between the depressed and non-depressed groups in terms of disease duration, percentage of females, duration of education, BMI, and medications. On the other hand, no differences were observed in Numano types, coronary, pulmonary, intracranial artery involvement, nor in the typical clinical symptoms in TA (Table 1).

**Table 1:** Clinical summary for TA patients with or without depression.

Variable	TA with depression (*n* = 12)	TA without depression (*n* = 28)	*p*-value
Age of onset, years	23.50 (19.25, 32.50)	37.00 (23.25, 42.50)	0.017*
Female, *n* (%)	12 (100.0)	25 (89.3)	0.332
Duration, months	45.00 (15.75, 156.00)	72.00 (24.00, 120.00)	0.738
Education, months	14.00 (12.00, 16.00)	13.00 (9.75, 14.00)	0.236
BMI (kg/m^2^)	23.03 (20.63, 26.59)	23.40 (21.51, 27.05)	0.738
*Medication paradigm, n (%)*			
Glucocorticoid	9 (75.0)	15 (53.6)	0.181
Prednisone equivalent, mg	10.00 (6.25, 15.00)	2.50 (0.00, 15.00)	0.260
cDMARDs	8 (66.7)	18 (64.3)	0.591
MTX	5 (41.7)	13 (46.4)	0.529
CTX	0 (0.0)	1 (3.6)	0.700
MMF	3 (25.0)	8 (28.6)	0.570
AZA	2 (16.7)	1 (3.6)	0.209
HCQ	0 (0.0)	2 (7.1)	0.485
btDMARDs	5 (41.7)	9 (32.1)	0.409
TCZ	4 (33.3)	7 (25.0)	0.430
ADA	1 (8.3)	1 (3.6)	0.515
Baricitinib	0 (0.0)	2 (7.1)	0.485
*Numano type, n (%)*			
I	2 (16.7)	2 (7.1)	0.346
IIa	0 (0.0)	1 (3.6)	0.700
IIb	2 (16.7)	10 (35.7)	0.207
III	0 (0.0)	0 (0.0)	
IV	0 (0.0)	1 (3.6)	0.700
V	8 (66.7)	14 (50.0)	0.268
Coronary artery	4 (33.3)	14 (50.0)	0.268
Pulmonary artery	4 (33.3)	6 (21.4)	0.337
Intracranial artery	5 (41.7)	12 (42.9)	0.612

BMI, body mass index; cDMARD, conventional synthetic disease-modifying antirheumatic drugs; btDMARDs, biologic disease-modifying antirheumatic drugs; MTX, methotrexate; CTX, cyclophosphamide; MMF, Mycophenolate mofetil; AZA, azathioprine; HCQ, hydroxychloroquine; TCZ, tocilizumab; ADA, adalimumab; * represents *p*-value < 0.05.

### Laboratory indicators for TA patients with or without depression

In the comparison of laboratory indicators, the authors found that the depressed group in TA had higher levels of WBC, NEUT, PLT, ESR, and hsCRP than the non-depressed group (9.58 [6.92, 10.52] ×10^9^/L vs. 6.53 [4.99, 9.10]×10^9^/L, *p* = 0.036; 69.05 [63.83, 76.38]% vs. 60.80 [51.33, 68.68]%, *p* = 0.033; 291.00 [232.50, 443.25]×10^9^/L vs. 231.00 [208.00, 278.75]×10^9^/L, *p* = 0.024; 16.00 [10.00, 58.50]mm/H vs. 7.50 [4.50, 17.75]mm/H, *p* = 0.013; 7.60 [2.32, 46.52]mg/L vs. 0.71 [0.32, 4.37]mg/L, *p* = 0.001), while the level of Hb was lower in the depressed group (104.50 [97.25, 126.00]g/L vs. 124.00 [115.50, 133.00]g/L, *p* = 0.009). The authors also compared the indexes of activity between the two groups. The levels of NIH and ITAS.A suggested significant differences (2.00 [2.00, 3.00] vs. 1.00 [0.00, 2.00], *p* = 0.007; 7.00 [4.00, 9.50] vs. 1.50 [0.00, 5.75], *p* = 0.012), while the level of ITAS2010 also emerged on a trend toward higher in the depressed group despite *p* > 0.05 (4.00 [3.25, 6.50] vs. 1.00 [0.00, 5.00], *p* = 0.056) (Table 2).

**Table 2:** Laboratory indicators for TA patients with or without depression.

Variable	TA with depression (*n* = 12)	TA without depression (*n* = 28)	*p*-value
WBC (×10^9^/L)	9.58 (6.92, 10.52)	6.53 (4.99, 9.10)	0.036*
NEUT (%)	69.05 (63.83, 76.38)	60.80 (51.33, 68.68)	0.033*
LYM (%)	24.60 (20.50, 32.88)	30.20 (24.15, 36.20)	0.172
Hb (g/L)	104.50 (97.25, 126.00)	124.00 (115.50, 133.00)	0.009**
PLT (×10^9^/L)	291.00 (232.50, 443.25)	231.00 (208.00, 278.75)	0.024*
Cr (μmol/L)	54.25 (45.45, 65.00)	56.30 (52.10, 64.58)	0.328
ALT (U/L)	21.00 (9.00, 28.75)	14.00 (10.00, 24.00)	0.570
ESR (mm/H)	16.00 (10.00, 58.50)	7.50 (4.50, 17.75)	0.013*
hsCRP (mg/L)	7.60 (2.32, 46.52)	0.71 (0.32, 4.37)	0.001**
IgA (g/L)	2.78 (1.82, 3.69)	2.23 (1.47, 2.92)	0.202
IgG (g/L)	9.69 (8.57, 13.11)	12.01 (10.33, 14.91)	0.074
IgM (g/L)	1.42 (0.96, 1.66)	1.06 (0.85, 1.51)	0.328
C3 (g/L)	1.31 (0.95, 1.53)	1.18 (1.00, 1.38)	0.342
C4 (g/L)	0.24 (0.18, 0.32)	0.24 (0.14, 0.31)	0.694
NIH	2.00 (2.00, 3.00)	1.00 (0.00, 2.00)	0.007**
ITAS2010	4.00 (3.25, 6.50)	1.00 (0.00, 5.00)	0.056
ITAS.A	7.00 (4.00, 9.50)	1.50 (0.00, 5.75)	0.012*

Numano type is divided according to Hata and Numano’s criteria; WBC, white blood cell count; NEUT, neutrophil count; Hb, hemoglobin; PLT, platelet count; ALT, alanine aminotransferase; Cr, creatinine; ESR, erythrocyte sedimentation rate; hsCRP, high-sensitivity C-reactive protein; C3, complement component 3; C4, complement component 4; Ig, immunoglobulin; * represents *p*-value < 0.05,** represents *p*-value < 0.010.

### The differences in cytokine profiles between TA patients and healthy controls

**Figure 1:**
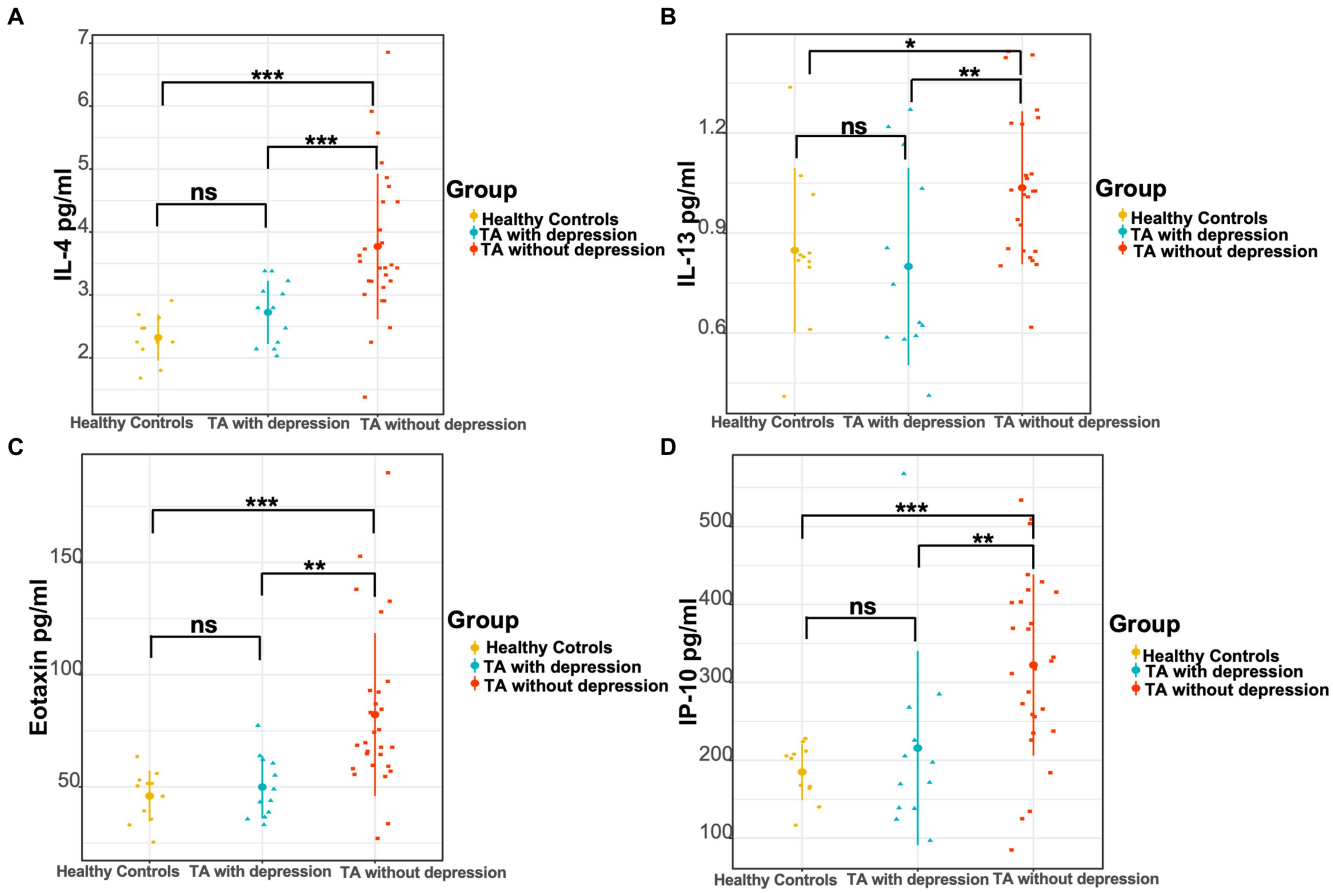
Comparison of three groups of cytokine levels. **(A)** The levels of IL-4, **(B)** IL-13, **(C)** eotaxin, and **(D)** IP-10 in the TA with depression, TA without depression, and healthy controls, respectively. *p < 0.05; **p < 0.01; ***p < 0.001.

As in previously known studies, many cytokines were elevated in TA patients compared to healthy controls, which was consistent with the inflammatory essence of the disease. The levels of IL-1rα, IL-2, IL-4, IL-6, IL-8, IL-12 (p70), eotaxin, FGF basic, G-CSF, IP-10, MCP-1, MIP-1α, and TNF-α were significantly higher in TA patients than the levels of 13 cytokines in the healthy controls (Table 3). No heterogeneity was found in the other 14 cytokines between TA patients and controls in our study. The authors further compared the cytokine profiles between depressed and non-depressed groups in TA patients. The levels of IL-4, IL-13, eotaxin, and IP-10 were significantly lower in the depressed group than in the non-depressed group (2.80 [2.17, 3.18]pg./ml vs. 3.51 [3.22, 4.66]pg./ml, *p* < 0.001; 0.66 [0.60, 1.12]pg./ml vs. 1.04 [0.82, 1.25]pg./ml, *p* = 0.008; 46.48 [37.06, 61.75]pg./ml vs. 69.14 [59.30, 92.80]pg./ml, *p* = 0.001; 184.50 [138.23, 257.25]pg./ml vs. 322.32 [241.98, 412.60]pg./ml, *p* = 0.005). However, there were no significant differences between the depressed TA group and healthy controls in the level of IL-4, IL-13, eotaxin, IP-10 (2.80 [2.17, 3.18]pg./ml vs. 2.25 [2.14, 2.64]pg./ml, *p* = 0.091; 0.66 [0.60, 1.12]pg./ml vs. 0.82 [0.82, 1.04]pg./ml, *p* = 0.487; 46.48 [37.06, 61.75]pg./ml vs. 50.45 [35.63, 53.05]pg./ml, *p* = 0.608; 184.50 [138.23, 257.25]pg./ml vs. 202.44 [163.94, 211.78]pg./ml, *p* = 0.976). In contrast, the levels of IL-4, IL-13, eotaxin, and IP-10 were significantly higher in the non-depressed TA group than in the healthy control group (3.51 [3.22, 4.66]pg./ml vs. 2.25 [2.14, 2.64]pg./ml, *p* < 0.001; 1.04 [0.82, 1.25]pg./ml vs. 0.82 [0.82, 1.04]pg./ml, *p* = 0.015; 69.14 [59.30, 92.80]pg./ml vs. 50.45 [35.63, 53.05]pg./ml, *p* < 0.001; 322.32 [241.98, 412.60]pg./ml vs. 202.44 [163.94, 211.78]pg./ml, p < 0.001) (Figure 1 and Table 3).

**Table 3:** Baseline levels of cytokines and chemokines in TA patients with depression, without depression, and healthy controls.

	Healthy controls (*n* = 11)	TA patients (*n* = 40)	TA with depression (*n* = 12)	TA without depression (*n* = 28)	*p*1	*p*2	*p*3	*p*4
IL-1β (pg/mL)	1.27 (1.18, 1.37)	1.37 (1.18, 1.77)	1.37 (1.18, 1.74)	1.42 (1.18, 1.91)	0.221	0.449	0.221	0.631
IL-1rα (pg/mL)	274.33 (203.45, 335.63)	390.90 (349.45, 489.60)	390.90 (349.45, 460.05)	416.39 (349.45, 534.71)	<0.001***	<0.001***	<0.001***	0.457
IL-2 (pg/mL)	0.73 (0.00, 1.10)	1.46 (0.73, 2.51)	1.64 (1.10, 1.82)	1.10 (0.35, 2.51)	0.034*	0.032*	0.078	0.694
IL-4 (pg/mL)	2.25 (2.14, 2.64)	3.35 (2.91, 3.98)	2.80 (2.17, 3.18)	3.51 (3.22, 4.66)	<0.001***	0.091	<0.001***	<0.001***
IL-5 (pg/mL)	26.02 (17.77, 73.84)	58.11 (26.02, 88.45)	60.02 (36.16, 92.32)	58.11 (13.59, 82.61)	0.353	0.069	0.747	0.224
IL-6 (pg/mL)	1.34 (0.39, 1.94)	7.54 (3.30, 14.60)	7.92 (4.55, 22.61)	6.52 (3.10, 14.06)	<0.001***	<0.001***	<0.001***	0.439
IL-7 (pg/mL)	1.93 (0.00, 7.17)	2.68 (1.93, 7.17)	1.93 (1.93, 4.75)	4.75 (1.93, 7.17)	0.214	0.740	0.149	0.154
IL-8 (pg/mL)	7.91 (7.91, 11.73)	13.60 (8.56, 20.29)	9.20 (7.59, 15.30)	13.76 (9.84, 21.93)	0.014*	0.316	0.004**	0.084
IL-9 (pg/mL)	426.64 (401.70, 440.39)	431.99 (413.65, 468.08)	426.89 (422.31,4 57.73)	434.79 (401.95, 468.84)	0.243	0.211	0.346	0.942
IL-10 (pg/mL)	0.00 (0.00, 0.00)	0.00 (0.00, 0.00)	0.00 (0.00, 0.08)	0.00 (0.00, 0.00)	0.088	0.190	0.396	0.570
IL-12p70 (pg/mL)	2.54 (2.54, 3.53)	3.53 (2.54, 6.22)	3.53 (2.54, 4.51)	4.02 (2.54, 6.46)	0.029*	0.091	0.040*	0.550
IL-13 (pg/mL)	0.82 (0.82, 1.04)	1.04 (0.82, 1.25)	0.66 (0.60, 1.12)	1.04 (0.82, 1.25)	0.133	0.487	0.015*	0.008**
IL-15 (pg/mL)	0.00 (0.00, 0.00)	0.00 (0.00, 0.00)	0.00 (0.00, 0.00)	0.00 (0.00, 0.00)	0.280	0.525	0.747	0.694
IL-17 (pg/mL)	3.30 (0.18, 6.31)	4.53 (3.30, 6.87)	4.06 (2.73, 4.81)	4.81 (3.49, 9.20)	0.051	0.379	0.026*	0.065
Eotaxin (pg/mL)	50.45 (35.63, 53.05)	64.61 (54.78, 84.24)	46.48 (37.06, 61.75)	69.14 (59.30, 92.80)	0.002**	0.608	<0.001***	0.001**
FGF basic (pg/mL)	18.79 (18.79, 21.42)	23.89 (20.13, 26.22)	21.42 (20.13, 23.89)	23.89 (19.45, 26.22)	0.042*	0.134	0.050	0.172
G-CSF (pg/mL)	90.53 (82.32, 101.37)	138.57 (112.10, 183.98)	120.09 (108.09, 165.29)	150.35 (114.76, 236.15)	<0.001***	0.001**	<0.001***	0.260
GM-CSF (pg/mL)	0.68 (0.11, 1.40)	1.17 (0.45, 2.48)	1.29 (0.68, 2.48)	1.05 (0.42, 2.48)	0.161	0.169	0.246	0.716
IFN-γ (pg/mL)	4.82 (3.95, 7.73)	6.50 (4.82, 9.67)	5.66 (4.82, 7.32)	7.12 (4.93, 11.02)	0.121	0.525	0.078	0.130
IP-10 (pg/mL)	202.44 (163.94, 211.78)	270.27 (187.35, 395.70)	184.50 (138.23, 257.25)	322.32 (241.98, 412.60)	0.007**	0.976	<0.001*	0.005**
MCP-1 (pg/mL)	23.28 (16.55, 29.61)	31.06 (25.30, 44.00)	31.27 (26.27, 36.72)	30.96 (24.96, 48.67)	0.003**	0.011*	0.005**	0.805
MIP-1α (pg/mL)	2.12 (1.61, 2.82)	4.23 (3.22, 5.92)	4.09 (2.67, 5.04)	4.35 (3.25, 10.15)	<0.001***	0.002**	<0.001***	0.247
PDGF-BB (pg/mL)	363.45 (237.83, 433.46)	415.79 (312.23, 504.89)	469.12 (320.27, 582.11)	411.82 (304.91, 486.81)	0.131	0.104	0.223	0.422
MIP-1β (pg/mL)	221.12 (215.93, 235.72)	236.19 (223.85, 249.95)	237.57 (226.79, 256.82)	236.19 (220.12, 245.60)	0.140	0.104	0.246	0.550
RANTES (pg/mL)	7581.99 (6942.25, 8470.32)	8178.82 (7689.31, 9070.81)	7987.64 (7702.62, 8887.28)	8265.12 (7583.14, 9209.47)	0.074	0.169	0.089	0.631
TNF-α (pg/mL)	46.57 (42.61, 49.20)	51.39 (46.68, 58.57)	50.51 (46.68, 54.65)	52.70 (46.79, 61.05)	0.015*	0.051	0.020*	0.439
VEGF (pg/mL)	0.00 (0.00, 0.00)	0.00 (0.00, 52.66)	0.00 (0.00, 0.00)	0.00 (0.00, 65.88)	0.054	0.525	0.124	0.475

p1: TA patients vs. healthy controls.

p2: TA with depression vs. healthy controls.

p3: TA without depression vs. healthy controls.

p4: TA with depression vs. TA without depression.

IL, interleukin; IFN-γ, interferon gamma; IP-10, interferon gamma-induced protein-10; FGF basic, fibroblast growth factor basic; G-CSF, granulocyte colony-stimulating factor; GM-CSF, granulocyte macrophage colony-stimulating factor; MCP-1, monocyte chemotactic protein-1; MIP-1α, macrophage inflammatory protein-1alpha; MIP-1β, macrophage inflammatory protein-1 beta; PDGF-BB, platelet-derived growth factor-BB; RANTES, regulated upon activation, normal T cells expressed, and secreted; TNF-α, tumor necrosis factor-alpha; VEGF, vascular endothelial growth factor. *represents *p*-value < 0.05,** represents *p*-value < 0.010, *** represents *p*-value < 0.001.

### Correlation between self-rating depression scale and cytokines, ESR, hsCRP, and disease activity

**Figure 2:**
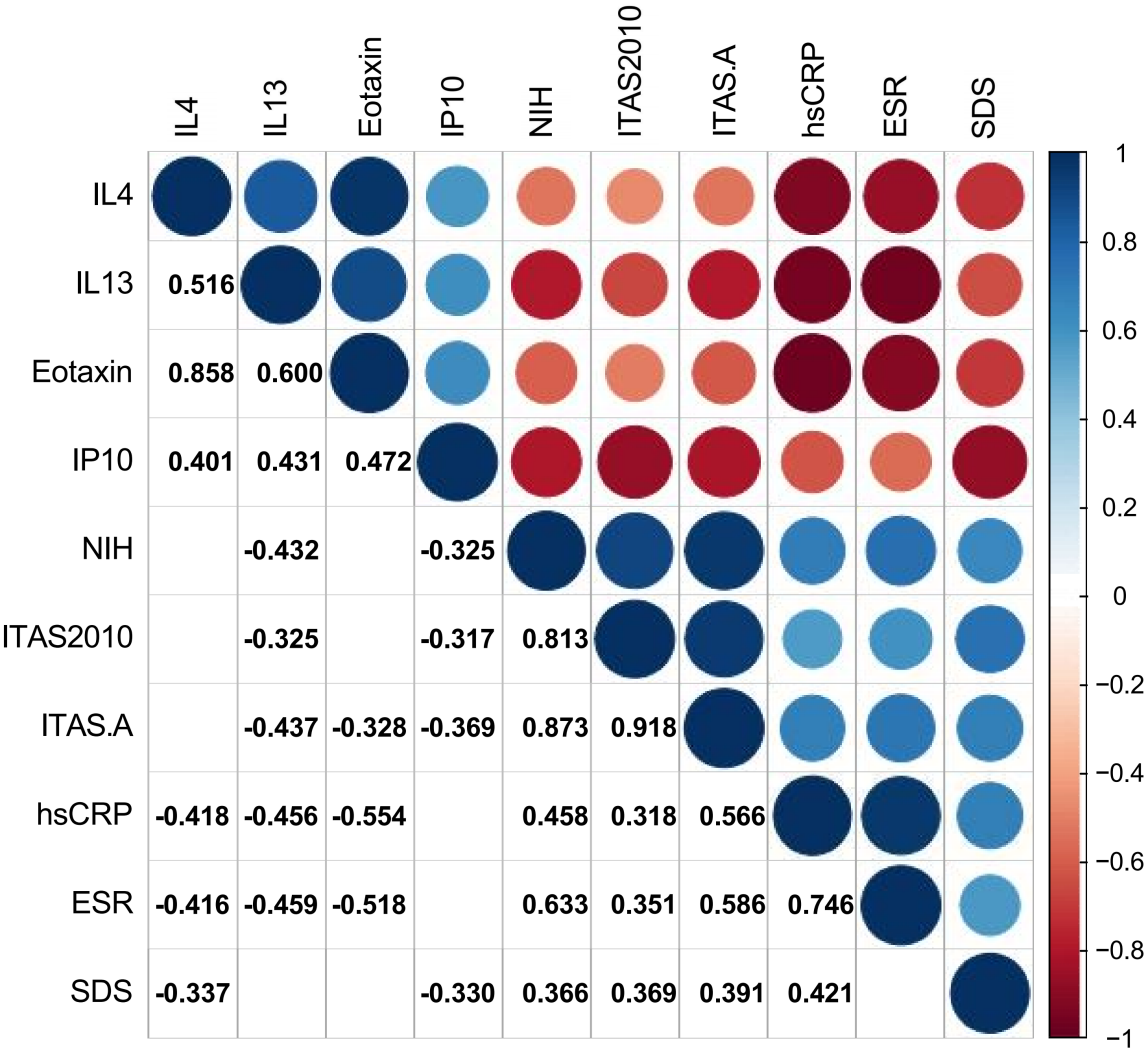
Correlations between cytokines, ESR, hsCRP, disease activity, and Self-Rating Depression Scale. The number and color in each box represented the correlation coefficient for two covariates corresponding to the box, of which blank indicated a non-statistically significant association.

The authors used Spearman’s test as appropriate to assess the correlations between SDS and other significant variables in TA patients. The authors presented statistically correlated parameters in Figure 2 (*p*-value < 0.05) and the correlation coefficient r was shown in the form, while the blank indicated no statistical association. SDS was negatively correlated with the levels of IL-4 and IP-10 (*r* = −0.337, *p* = 0.033; −0.330, *p* = 0.038), while positively correlated with hsCRP, NIH, ITAS.A and ITAS2010 (*r* = 0.421, *p* = 0.007; *r* = 0.366, *p* = 0.020; *r* = 0.391, *p* = 0.013; *r* = 0.369, *p* = 0.019, respectively). In the meantime, the levels of IL-4, IL-13 and eotaxin were negatively associated with ESR (*r* = −0.416, *p* = 0.008; *r* = −0.459, *p* = 0.003; *r* = −0.518, *p* = 0.001) and hsCRP (*r* = −0.418, *p* = 0.007; *r* = −0.456, *p* = 0.003; *r* = −0.554, *p* < 0.001).

### Risk factors of depression in TA patients

To identify independent predictors of depression in TA, the authors use multivariate logistic regression analysis to confirm the value of cytokines, which were significantly different in univariate analysis (*p* < 0.05) (Table 4). The univariate logistic regression analysis showed that age of onset and the levels of IL-4, IL-13, eotaxin, and IP-10 were protective in the depressed TA group (OR [95% CI] 0.926 [0.864, 0.992], *p* = 0.028; 0.176 [0.045, 0.689], *p* = 0.013; 0.021 [0.001, 0.555], *p* = 0.021; OR [95% CI] 0.932 [0.883, 0.983], *p* = 0.010; 0.992 [0.985, 0.999], p = 0.028, respectively), while ESR and NIH, ITAS.A as potential risk factors (OR [95% CI] 1.053 [1.004, 1.104], *p* = 0.032; 2.654 [1.251, 5.631], *p* = 0.011; OR [95% CI] 1.246 [1.035, 1.500], *p* = 0.020, respectively). In order to exclude confounding factors and build a multivariate regression model to better identify risk factors in the depressed group, the authors included traditional demographic risk factors, adjusted for gender, education, and disease duration. The results suggested that both the lower level of IL-4 and age of onset were risk factors for depression in TA patients (OR [95% CI] 0.124 [0.018, 0.827], *p* = 0.031; 0.870 [0.765, 0.990], *p* = 0.035, respectively).

**Table 4:** Multiple logistic regression model with cytokines and confounding factors.

	Univariable analysis		Multivariable analysis	
	Odds ratio (95% CI)	*p*-value	Odds ratio (95% CI)	*p*-value
Age of onset	0.926 (0.864, 0.992)	0.028*	0.870 (0.765, 0.990)	0.035*
Female		0.999		
Disease duration	0.999 (0.999, 1.005)	0.716		
Education time	1.170 (0.941, 1.456)	0.158		
ESR	1.053 (1.004, 1.104)	0.032*		
hsCRP	1.095 (0.984, 1.219)	0.096		
NIH	2.654 (1.251, 5.631)	0.011*		
ITAS.A	1.246 (1.035, 1.500)	0.020*		
ITAS2010	1.187 (0.967, 1.457)	0.102		
IL-4	0.176 (0.045, 0.689)	0.013*	0.124 (0.018, 0.827)	0.031*
IL-13	0.021 (0.001, 0.555)	0.021*		
Eotaxin	0.932 (0.883, 0.983)	0.010*		
IP-10	0.992 (0.985, 0.999)	0.028*		

*represents *p*-value < 0.05.

## Discussion

In this research, the authors recorded the levels of 27 cytokines, laboratory, and clinical data in patients with Takayasu arteritis (TA). Our study has reported the age of onset and IL-4 were risk factors for depressed TA patients and showed the depression group was more active in the disease for the first time. The authors found that depression was correlated with lower levels of IL-4, IL-13, eotaxin, and IP-10, suggesting the abnormal immune responses contributing to the evolution of depression in TA.

The authors found the depressed group was more active in TA patients. There were higher levels of ESR, hsCRP, NIH, ITAS.A, and ITAS2010 in TA with depression compared with non-depression. Previous studies have found depression and inflammation are intertwined, which depression facilitates inflammatory responses and inflammation promotes depression (21). Due to the inflammatory properties of autoimmune disease, many research studies devoted to studying the bidirectional inflammatory pathway between autoimmune diseases and depression (22). Imran et al. (23) identified a positive correlation between the severity of depression and the disease activity in rheumatoid arthritis (RA). Similar to other autoimmune disorders such as Behçet disease and rheumatoid arthritis (23, 24), the disease activity also showed a positive correlation with depression scores in TA (11, 25), which was consistent with our results.

Our study then compared the clinical characterizations between the depressed and non-depressed groups in TA patients. There was no heterogeneity in disease duration, BMI, educational time, and the female ratio. The younger age of onset was identified as an independent risk factor for depression in TA patients. It was shown that the younger age had well-known associations with depression (26). This can also be observed in autoimmune diseases. Previous research has shown that younger age in people under 40 years old had a significant interaction with RA in terms of depression risk (27, 28). In the existing life quality reports on TA, it was argued that age was negatively associated with emotional scores (29). However, studies on the relationship between age in TA patients with depression are lacking. Consistent with RA, the authors found that the younger age of onset showed a significantly positive correlation with depression in TA. This may be related to the fact that young patients are often actively involved in social work. The physical discomfort of TA combined with work stress can have a negative impact on the individual and trigger negative emotions.

The coexistence of immune-mediated inflammatory diseases and depression has long been recognized (7). Therefore, the authors focused on the analysis of the link between cytokines and depression in TA. In our study, the authors found the levels of IL-4, IL-13, eotaxin, and IP-10 were significantly lower in the depression of TA patients. Furthermore, the lower level of IL-4 proved to be an independent risk factor for depressed TA patients. IL-4 is an important anti-inflammatory factor produced by activated Th2 cells, thereby reducing the inflammatory response (30). Previous studies have proved that deficiencies in IL-4 can lead to an inability to recover in depressed mice caused by stress (31). It was shown that IL-4 reprogrammed the microglia, which activated the brain-derived neurotrophic factor signaling pathways to protect hippocampal nerves in depressed mice (32). These results indicated that IL-4 exerted neuroprotective function to fight depression. Current research found an elevated level of IL-4 in depressed populations and rats after exposure to chronic mild stress (33, 34) to suppress the inflammatory response. Recent findings suggested that IL-4 and IL-13 might play a significant role in the downregulation of inflammatory processes underlying RA pathology and beneficially modulate the course of the disease (35). In autoimmune disease, scholars have found that an increase in the ratio of Th1/Th2 cells could promote the production of depressive symptoms in collagen-induced arthritis mice (36). Hyperactive or hypoactive stress systems through modulation of the Th1/Th2 cytokine balance might be associated with abnormalities of the “systemic anti-inflammatory feedback,” contributing to the pathogenesis of depression and autoimmune diseases (37). This can explain that a decrease in IL-4 may indicate a lower proportion of Th2 cells, leading to an increase in the Th1/Th2 ratio, which can lead to TA patients with depression. It was shown that TA patients had higher serum levels of IL-4 and IL-13 than healthy controls. However, Gao et al. (38), in their investigation of Th2 cell characteristics in TA, identified there was not a significant difference in IL-4 between active and inactive groups. This aligns with our discovery that IL-4 levels did not show a correlation with NIH/ITAS.A/ITAS2010, which implies that the reduction of IL-4 in TA patients with depression might be specific to this condition.

The cytokine IL-13 is grouped together with IL-4 as anti-inflammatory cytokines, both of which are closely linked in the genome (39, 40). However, IL-13 was proven only one-tenth of the inhibiting effect of IL-4 in RA (41), without a direct correlation with depression. A cross-sectional study on depression in patients with breast cancer identified that the level of IL-13 was lower in the depressed group than in the non-depressed (13), a lack of further research to clarify the pathological mechanisms. Previous studies have found that IL-13 is significantly elevated in TA (38). Our result that the lower level of IL-13 was associated with depression might be related to the synergy of IL-4 reduction, with the need for further research to prove it. Eotaxin is a potent eosinophil chemoattractant cytokine (42). Its main receptor, the CC chemokine receptor 3 (43–45), is expressed on Th2 lymphocytes, implicated in the production of the Th2 cytokines (IL-4, IL-13) (46). Consequently, eotaxin has been proven to tend to a Th2 response (47). However, the current research studies remains contradictory about the role of eotaxin in depression (46). In previous studies, eotaxin was found to be higher in depressed patients than in healthy controls, but still remained elevated from baseline after 8 weeks of effective treatment (48). The determination of chemokines found no significant difference in eotaxin between TA and healthy controls (49). The authors confirmed that the lower level of eotaxin had a suggestive effect on TA patients with depression to a certain extent, which might be consistent with the declined IL-4. IP-10 is a Th1-related chemokine, secreted by T lymphocytes, NK cells, and monocytes (50) and demonstrated to be associated with multiple rheumatic immune disease activity (51). It was known that the HIV^+^ subjects with depression had increased levels of IP-10 compared to their non-depressed counterparts (52). Similar findings have not been identified in autoimmune diseases. The authors first found the declined level of IP-10 attributed to the depression in TA, the mechanism remaining a mystery.

Given the rarity of TA, our study is limited by a small sample size. Additionally, being a retrospective and cross-sectional study, it lacks a prospective plan and a follow-up cohort. In future, the authors will expand the number of patients and collect samples from patients with other systemic inflammatory diseases for more rigorous research.

## Conclusion

This study presented that the serum levels of IL-4 were significantly decreased in TA patients with depression. It suggests that IL-4 may play an important part in the development of depression in TA patients. Clinicians need to be vigilant about the decline of IL-4 in order to predict the potential risk of depression in TA patients and respond positively.

## Data availability statement

The raw data supporting the conclusions of this article will be made available by the authors, without undue reservation.

## Ethics statement

The studies involving humans were approved by the Ethics Committee of Beijing Anzhen Hospital (Approval Number: 2023183X). The studies were conducted in accordance with the local legislation and institutional requirements. The participants provided their written informed consent to participate in this study. Written informed consent was obtained from the individual(s) for the publication of any potentially identifiable images or data included in this article.

## Author contributions

YZ: Conceptualization, Data curation, Formal analysis, Investigation, Methodology, Validation, Visualization, Writing – original draft. SY: Data curation, Writing – original draft. AF: Investigation, Methodology, Writing – original draft. JD: Data curation, Writing – original draft. NG: Funding acquisition, Validation, Writing – review & editing. LP: Conceptualization, Funding acquisition, Writing – review & editing. TL: Conceptualization, Data curation, Funding acquisition, Validation, Writing – review & editing.

## Glossary

**Table:** 

TA	Takayasu arteritis
SDS	The Zung Self-Rating Depression Scale
OR	Odds ratio
CI	Confidence interval
BMI	Body mass index
NIH	National Institutes of Health criteria
ITAS	Indian Takayasu’s Arteritis Activity Score
ITAS.A	ITAS with acute-phase reactants
ALT	Alanine aminotransferase
CR	Creatinine
ESR	Erythrocyte sedimentation rate
hsCRP	High-sensitivity C-reactive protein
IL	Interleukin
IFN-γ	Interferon gamma
IP-10	Interferon gamma-induced protein-10
FGF basic	Fibroblast growth factor basic
G-CSF	Granulocyte colony-stimulating factor
GM-CSF	Granulocyte macrophage colony-stimulating factor
MCP-1	Monocyte chemotactic protein-1
MIP-1α	Macrophage inflammatory protein-1alpha
MIP-1β	Macrophage inflammatory protein-1 beta
PDGF-BB	Platelet-derived growth factor-BB
RANTES	Regulated upon activation, normal T cells expressed, and secreted
TNF-α	Tumor necrosis factor-alpha
VEGF	Vascular endothelial growth factor
C3	Complement component 3
C4	Complement component 4
Ig	Immunoglobulin
WBC	White blood cell
RBC	Red blood cell
NEUT	Neutrophil count
PLT	Platelet count
cDMARD	Conventional synthetic disease-modifying antirheumatic drugs
btDMARDs	Biologic disease-modifying antirheumatic drugs
MTX	Methotrexate
CTX	Cyclophosphamide
MMF	Mycophenolate mofetil
AZA	Azathioprine
HCQ	Hydroxychloroquine
TCZ	Tocilizumab
ADA	Adalimumab
